# Effects of Midsole Hardness on the Mechanical Response Characteristics of the Plantar Fascia during Running

**DOI:** 10.3390/bioengineering10050533

**Published:** 2023-04-27

**Authors:** Xiaolan Zhu, Jiaojiao Liu, Hui Liu, Jingxi Liu, Yufeng Yang, Haichun Wang

**Affiliations:** 1 Sport Science School, Beijing Sport University, Beijing 100084, China; 2 School of Naval Architecture and Ocean Engineering, Huazhong University of Science and Technology, Wuhan 430074, China

**Keywords:** foot–shoe model, finite-element method, plantar fascia, midsole hardness, biomechanics

## Abstract

High long-term stress on the plantar fascia (PF) is the main cause of plantar fasciitis. Changes in the midsole hardness (MH) of running shoes are an important factor leading to the alteration of the PF. This study aims to establish a finite-element (FE) model of the foot–shoe, and investigates the effects of midsole hardness on PF stress and strain. The FE foot–shoe model was built in ANSYS using computed-tomography imaging data. Static structural analysis was used to simulate the moment of running push and stretch. Plantar stress and strain under different MH levels were quantitatively analyzed. A complete and valid 3D FE model was established. With an increase in MH from 10 to 50 Shore A, the overall stress and strain of the PF were decreased by approximately 1.62%, and the metatarsophalangeal (MTP) joint flexion angle was decreased by approximately 26.2%. The height of the arch descent decreased by approximately 24.7%, but the peak pressure of the outsole increased by approximately 26.6%. The established model in this study was effective. For running shoes, increasing the MH reduces the stress and strain of PF, but also imposes a higher load on the foot.

## 1. Introduction

Plantar fasciitis is a common soft-tissue foot injury. In the United States, over 2 million individuals annually undergo treatment for this condition, accounting for 11–15% of medical visits related to foot pain [1]. Plantar fasciitis can occur in younger joggers, with an incidence as high as 10% [2]. It is the third common foot injury associated with running [3], and the main cause of heel pain [4,5]. The condition is painful and can affect the quality of the daily life of the patients.

Biomechanical factors represent the most common etiology of plantar fasciitis [4]. Previous studies revealed that high loads on the foot [6,7,8], the repetitive flexion and extension of the metatarsophalangeal (MTP) joint [9,10], and arch collapse [11,12] are important risk factors for plantar fasciitis. Midsole hardness (MH) is the key parameter with regard to the quality of running shoes. Excessively low or high MH causes abnormal changes in the biomechanical characteristics of the foot during running, causing foot-related injuries [13,14,15]. Peak plantar pressure increases with increasing MH [16]. Although running shoes with low hardness provide better cushioning and comfort, the stability control ability of the foot is poor [16,17,18,19]. An appropriate MH level can better maintain and support the arch, thereby preventing arch collapse. In addition, the MH of running shoes affects midsole flexibility, and determines the flexion stiffness of the midsole [20]. Some studies demonstrated that an increase in the flexion stiffness of the midsole at the MTP joint could lead to a decrease in the range of motion of this joint [20].

The MH effects of running shoes on the lower limbs and the risk factors for plantar fasciitis [6] suggest that this hardness indirectly affects the biomechanical characteristics of the plantar fascia (PF). Although some studies indicated that unsuitable shoes are one of the risk factors for plantar fasciitis [11,21,22,23], most risk factors are only speculated from the anatomical function of the PF and the biomechanical perspective owing to the limitations of experimental conditions. Research that directly confirms this inference is lacking. Due to the direct contact among running shoes, feet, and the ground, a suitable MH level can play a fundamental protective role and improve sports performance. In contrast, the long-term use of unsuitable running shoes can increase the risk of foot injuries [24].

Research on the influence of running shoes on the biomechanical characteristics of the foot has mainly adopted an experimental biomechanical method to analyze the external mechanical situation, overall motion, ground support reaction force, and planar pressure distribution of the foot and the shoe (insole) [14,15]. There are also new experimental methods such as new sensors [25] and algorithms [26] to explore the relationship between the foot and the shoe under different working conditions. It is difficult to comprehensively and directly explain the internal interaction mechanism between the foot and shoe or insole, time-consuming, and costly. The finite-element method (FEM) plays a key role in solving the above problems, as it can present all the internal mechanical information in the form of calculated equations through modeling analysis to predict the stress distribution load between the foot and different supports, and provide the internal stress- and strain-state information of the foot. With the development of the FEM, its application in the field of foot biomechanics is increasingly extensive. The foot–shoe coupling model is oversimplified, and only the main skeleton and peripheral tissues are retained [27], which may affect the simulation results.

This study aims to establish an accurate 3D FE model of the foot–shoe, and investigates the effects of the midsole hardness on PF stress and strain. This study provides a basis for subsequent research on foot–shoe biomechanics, and a visual platform for the functional verification of running shoes.

## 2. Methods

### 2.1. Subject and Shoes

A healthy male subject (age, 25 years; height, 168 cm; weight, 67 kg; length, 25.5 cm; width, 7.5 cm) who was an amateur runner participated in the present study. Following an inquiry and X-ray examination, the anatomical structure of their foot was determined as normal. The participant had no history of foot or lower-limb trauma, or surgery. The shoes used in the experiment were a pair of ordinary running shoes provided by Anta (China) Co., Ltd., Xiamen, China. The material used for the upper part of the shoe was space leather, the midsole was MD (EVA Foaming, 50 Shore A), and the outsole was rubber. The research protocol was approved by the ethics committee of Beijing Sport University (2021114H), and the participant read and signed an informed consent form before data collection.

### 2.2. Construction of the 3D FE Foot–Shoe Model

Two-dimensional images of the subject’s right foot while he wore the shoe were obtained using a computed-tomography scanner (GE Lightspeed 16-slice multislice spiral CT, Boston, USA). The layer was 1.25 mm thick, and the pixels were 512 × 512. During scanning, the foot was in a neutral position without weight bearing. Medical-image processing (Mimics 17.0, Materialise Inc., Leuven, Belgium) and interactive CAD/CAM System (UGNX 10.0, Siemens PLM Software Inc., Plano, TX, USA) software were used to construct the 3D geometric entity foot–shoe models. After that, FE package ANSYS 12.1 (Swanson Analysis, Houston, PA, USA) was used to assemble the foot–shoe components, create the FE mesh, and conduct subsequent validation and action simulation.

In the present study, bony components were embedded into the encapsulated soft-tissue volume using an embedded region constraint [28]. Similarly, the encapsulated soft tissues of the foot, midsole, outsole, and upper parts of the running shoes were assembled using this method. The ligaments and PF were defined by connecting the corresponding attachment points on the bones. The PF was divided into five sections linking the insertions between the calcaneus and MTP joints. The cartilage was modeled on the basis of its anatomical location to extract cartilage and lacunar solid elements, which were endowed with the material properties of the cartilage in order to simulate joint motion [29,30,31].

The material properties of each foot-tissue [32,33,34,35] and shoe [27,36] component were selected from the literature and are listed in Table 1. The material properties of the midsole, including the uniaxial tensile test parameters (stress–strain data curve), were obtained using the same material hardness as that of Shore A 10–50 in the relevant literature [37]. The curve-fitting function, defined using ANSYS 12.1 nonlinear hyperelastic material, was used to fit the material characteristics and the function model. The third-order Yeoh hyperelastic model [38] was selected to fit the characteristics. Strain energy density function W was defined as follows:

$$W=\overset{3}{\underset{i=1}{\sum }}C_{i0}{\left(\bar{I}-3\right)}^{i}+\sum_{i=0}^{3}\frac{1}{Di}{\left(J-1\right)}^{2i}$$

where $\bar{I}$ is the deviation invariant, *J* is the volume ratio, and *C* and *D* are the material attribute parameters.

Free-meshing technique was used to create the FE mesh. A cuboid with length of 300 mm, width of 136 mm, and height of 10 mm was constructed by establishing a local coordinate system on the sole surface to simulate the ground support plate. The bony soft-tissue structures and shoes were meshed using the SOLID187 element (a three-dimensional 10-node tetrahedral element with intermediate nodes), and the mesh size of each foot bone was 2–36 mm (proximal phalanges were 1 mm, the metatarsal was 10 mm, and the other bones were divided within the range of 10–36 mm according to the model structure). The mesh size of the peripheral tissue of the foot was 36 mm, the uppers were 40 mm, the midsole was 4 mm, the outsole was 2 mm, and the ground support plate was 2 mm. The ligaments and the PF were defined using the tension-only LINK 10 element (an element with bilinear stiffness matrix properties), which resulted in a total of 344,636 nodes and 442,512 elements.

### 2.3. FE Model Validation

The validity of the 3D FE foot–shoe model was the basis for subsequent biomechanical research. In the present study, biomechanical tests and FE simulations of the human foot in a shoe were conducted in the balanced standing position. The pressure/distribution data of the foot plantar pressure and sole obtained from the simulation calculation were compared with obtained results from actual tests to synchronously validate the foot–shoe coupling model. In addition, the present study aimed to apply the foot–shoe finite element model to study PF biomechanics. Accordingly, the results of the stress and strain simulation value of PF were extracted to further evaluate its validity.

#### 2.3.1. Finite-Element Simulation of a Balanced Standing Position

**Figure 1:**
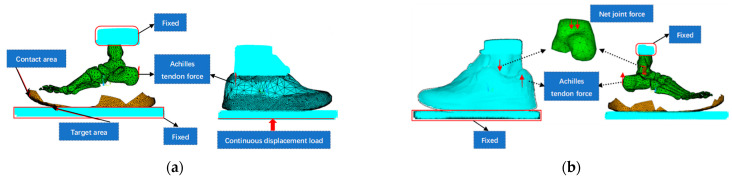
Boundary and load conditions for (**a**) foot–shoe model validation and (**b**) push-off during running.

When standing with both feet balanced, the vertical force for a person weighing 67 kg is approximately 335 N per foot, and this value is affected by the muscles of the feet and lower limbs. Through several simulations, a continuous displacement load (equivalent to a vertical force of 335 N) was vertically applied upward to the ground support plate to ensure that uniform action was exerted on the shoe outsole [31,39]. Regarding muscle force, the present study only considers the role of gastrocnemius muscle force [31,40], which is responsible for approximately 50% of the foot load (approximately 167.5 N) applied vertically upward to the attachment point of the Achilles tendon on the calcaneus. All of the nodes on the distal surface of the tibia and fibula were completely fixed, and the ground support plate only retained the freedom of the sagittal plane. The contact type was quasistatic contact analysis with nonlinear large deformation. The friction coefficient was 0.6 [41]. Overall, 4993 nodes were used to generate surface–surface contact elements between the shoe outsole and the ground support plate (Figure 1a).

#### 2.3.2. Foot and Sole Pressure/Distribution Data Testing 

A Pedar plantar pressure sensing insole measurement system (Pedar-X; Novel, Inc., Munchen, Germany; 100 Hz, 99 pressure sensors, pressure measurement range of 15–600 kPa) and Footscan plantar pressure measurement system (RSscan, Belgium; 200 Hz, 0.5 m in length, 4096 pressure sensors, pressure measurement range of 1–127 N/cm, which is approximately 10–1270 kPa) were used to collect the right foot plantar–outsole pressure data of the participant standing with both feet balanced. The collected metrics include plantar–outsole peak pressure and pressure distribution data, which were used to verify and analyze the validity of the model simulation results.

### 2.4. FE Simulation of the Mechanical Response of the PF to Midsole Hardness

#### 2.4.1. Load Acquisition Simulation Experiments 

The push-off instant (the moment when the heel leaves the ground) during running was selected for the simulation in the present study, and only the sagittal load of the foot was considered. Therefore, a high-speed eight-camera motion-capture system (Motion Analysis Corporation, Santa Rosa, CA, USA; 200 Hz) and 3D force plate (Kistler Instrumente AG, Winterthur, Switzerland; 1000 Hz) were used to synchronously determine the kinematic and dynamic foot parameters of the subject wearing shoes at push-off while running at a speed of 3.8 ± 0.2 m/s, and an infrared velocimeter ( Newtest Oy, Tyrnävä, Finland) was used to monitor the running speed. Further, the collected data were processed using Cortex 2.6.2 (Motion Analysis Cooperation, Santa Rosa, CA, USA). The net joint force and joint moment (plantar flexion moment) along the vertical axis of the ankle joint were calculated using the inverse dynamics method. The Achilles tendon tension (Fat) was defined as the ratio of the plantar flexion moment (Ma) of the ankle joint to the Achilles tendon force arm (armat).

The moment arm of the Achilles tendon was simplified to be the same as that of the last moment at which the foot was in neutral position, that is, the horizontal distance between the Achilles tendon attachment points and the center of the ankle joint, which was measured directly from CT images using the MIMICS 17.0 measuring tool. Ankle net force and Achilles tendon force data are shown in Table 2 (the vertical axis joint net force is positive upwards and negative downwards).

#### 2.4.2. Loading Mode and Hardness Parameter Setting

As shown in Table 2, net joint force was applied to the center of the ankle joint (middle part of the upper surface of the talus [42]), and Achilles tendon force was applied to the attachment point of the Achilles tendon via the node force. The ground support plate and upper ends of the tibia and fibula were completely fixed. The friction contact between sole and ground was defined (Figure 1b). Nonlinear large deformation quasistatic contact analysis was used to simulate the mechanical response characteristics of the PF at push-off while running with five levels of midsole hardness (Table 1).

### 2.5. Correlation Index Definition

Excessive stress on the PF is the main cause of plantar fasciitis. Comprehensive studies on the risk factors of plantar fasciitis have revealed that, when the foot is subjected to high or uneven force, the arch structure and function of the foot are abnormal, and the repetitive overflexion and extension of the MTP joint could exert abnormal stress levels on the PF [7]. Therefore, MTP joint flexion angle (∠α), arch-height, and plantar-pressure data were extracted to analyze the mechanical mechanism of plantar fasciitis induced by a change in midsole hardness.

Changes in ∠α and arch height are indirectly expressed as the displacement of the corresponding vertical axis of the skeleton in the foot. The change in ∠α was determined by defining relative displacement distance ∆L between the proximal phalangeal end and proximal MTP end on the vertical axis. The change in arch height was determined by defining the relative displacement distance ∆H between the medial cuneiform bone of the foot and the vertical axis of the calcaneus.

## 3. Results

### 3.1. Validation of the FE Foot–Shoe Model

**Figure 2:**
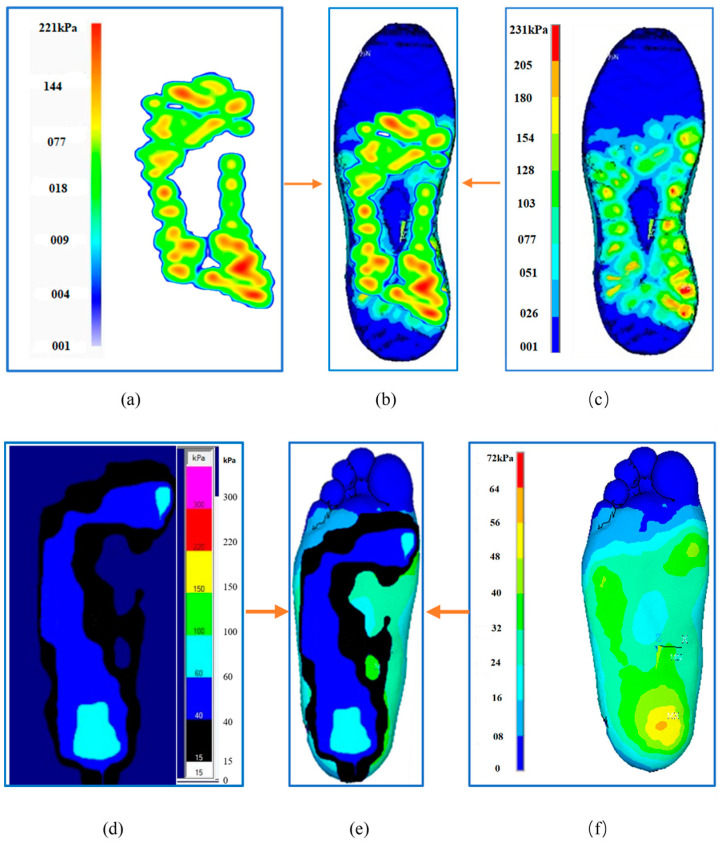
Simulated and measured (**a**–**c**) outsole pressure and (**d**–**f**) plantar foot pressure. (**a**) Measured outsole pressure; (**b**) comparison diagram; (**c**) simulated outsole pressure; (**d**) measured plantar pressure; (**e**) comparison diagram; (**f**) simulated plantar pressure.

As shown in Figure 2a–c, the simulated outsole peak pressure (231 kPa) was larger than the actual measured value by 10 kPa (221 kPa), representing a difference of approximately 4.33%. The simulated whole contact area (approximately 70 cm^2^) was smaller than the measured value by 2 cm^2^ (72 cm^2^), representing a difference of approximately 2.8%. Plantar foot pressure data (Figure 2d–f) illustrate that the simulated peak plantar pressure (64 kPa) was 20% smaller than the measured value (80 kPa). The entire simulated distribution area (approximately 131 cm^2^ in the part with pressure >15 kPa) was larger than the measured area by 20 cm^2^ (approximately 111 cm^2^), representing a difference of approximately 18%. When the pressure distribution cloud maps were compared (Figure 2b,e), the pressure distribution of the simulated and measured soles was consistent, and they were mainly located in the middle of the foot and heel. The distribution of the simulated and measured plantar pressure was consistent, with the minimum at the toe, the middle at the sole and arch of the foot, and the maximum at the heel.

**Figure 3:**
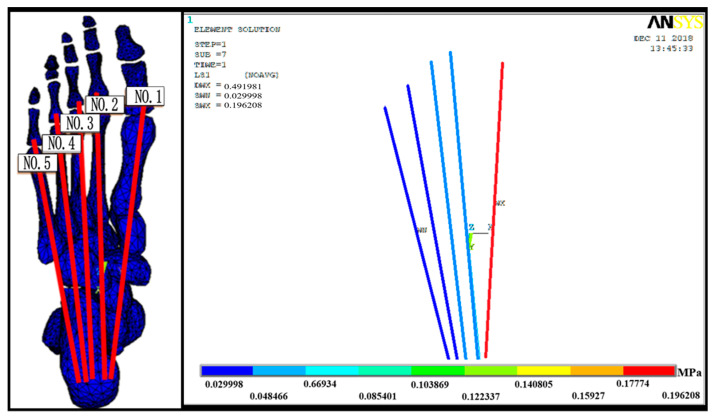
Von Mises stress distribution in the plantar fascia.

PF stress–strain results illustrate that the stress decreased consecutively from the first to the fifth bundle of the plantar fascia, i.e., from the medial to the lateral side (Figure 3). The stress and strain for the first bundle (medial) were approximately 0.196 MPa and 0.056%, respectively, which were the largest values, whereas those of the fifth bundle (lateral) were approximately 0.029 MPa and 0.009%, respectively.

### 3.2. Effects of Midsole Hardness on the PF and Its Mechanical Mechanism

#### 3.2.1. PF Stress–Strain Results 

The stress distribution was the same for the five PF bundles under different levels of midsole hardness. The stress value of the five PF bundles decreased from the first (medial) to the fifth bundle (lateral). The stress range of the five PF bundles was 0.809–2.300 MPa, and the strain range was between 0.23% and 0.66% (Table 3). A summation of stress on the five PF bundles was used to represent the overall level of PF stress, which decreased by 0.112 MPa, approximately 1.62%, as the hardness was increased from 10 to 50.

#### 3.2.2. Mechanical Response Results of Risk Factors for Plantar Fasciitis

**Figure 4:**
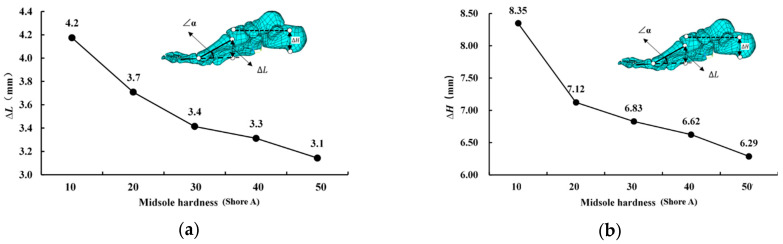
Relative displacement distance (**a**) ΔL and (**b**) ΔH curves at different levels of midsole hardness.

As ∆L increased, ∠α increased. The change in arch height increased as ∆H increased. Therefore, both ∆L and ∆H decreased with the increase in midsole hardness. Specifically, as the midsole hardness increased from 10 to 50, ∆L decreased by approximately 1.1 mm (approximately 26.2%; Figure 4a), and ∆H decreased by approximately 2.06 mm (approximately 24.7%; Figure 4b).

**Figure 5:**
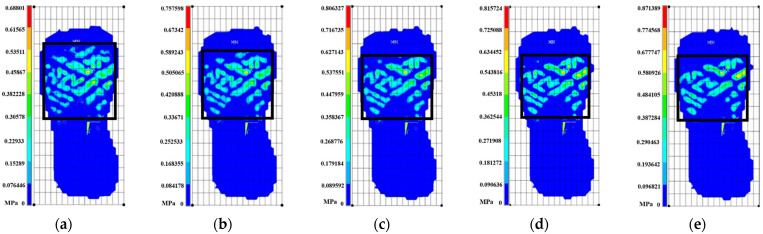
(**a**–**e**) Outsole pressure at different levels of midsole hardness. (**a**) Shore A10; (**b**) Shore A20; (**c**) Shore A30; (**d**) Shore A40; (**e**) Shore A50.

In addition, plantar pressure was indirectly expressed as outsole pressure. As shown in Figure 5, to distinguish the pressure distribution range of the outsole at different levels of midsole hardness, the entire ground support plate was divided into 256 grids of the same size. For example, when the midsole hardness was 10, the distribution range of the sole pressure occupied approximately 60 grids, whereas when midsole hardness was 50, it occupied approximately 40 grids, representing an overall decrease of approximately 33.3%. Peak pressure increased as midsole hardness increased. When midsole hardness was 10, peak pressure was approximately 0.688 MPa, which was the lowest value, and when midsole hardness is 50, pressure was approximately 0.871 MPa, which was the highest value; the peak pressure of the outsole increased by approximately 0.183 MPa (approximately 26.6%).

## 4. Discussion

### 4.1. Validity of 3D Finite-Element Foot–Shoe Model

The FE foot–shoe model exhibited high geometric similarity to the actual foot. In predicting the mechanical characteristics, the simulation results of outsole pressure were similar to the measured results. The observed differences for the distribution area and peak pressure may have been related to the difference between the Footscan plantar pressure measurement system, and finite-element simulation measurement range and accuracy. There was a certain deviation in the prediction of foot plantar pressure, and the range of the simulated plantar pressure distribution results was larger than that of the measured results. The main reason for this difference was that the foot–insole was not completely fitted during the actual measurement process, unlike that in the FE simulation process; therefore, the simulation results encompassed a broader range of pressure distribution despite the relatively low peak pressure. In addition, this could have been related to the definition of the material properties of the model. The definition of the foot–sole of the material in the present study was the isotropic linear material except the midsole, and there was a deviation [43] in the process of force transmission. However, on the basis of measured and simulated values obtained in several prior studies [40,44,45], the deviation rates ranged from 0.7% to 25%, and our results were within this deviation range, indicating that our model was valid.

Regarding the mechanical characteristics of the PF, the results illustrate that the stress and strain of the PF in a standing state with both feet balanced decreased consecutively from the inside to the outside of the foot, which is consistent with the mechanical test of PF materials [46] and the relevant results of the finite-element simulation [45]. The simulated plantar-fascia stress during shoe wearing was slightly less than that reported by Hsu et al. [40] during balanced barefoot standing. This difference may have been due to the fact that shoes absorb some of the forces acting directly on the foot through the deformation of the sole itself, resulting in relatively low simulated stress on the fascia compared to that with bare feet. Conversely, this finding may have also been related to the contact between the insole and foot, which could support the arch of the foot and thus reduce the traction of the fascia. On the basis of the strain results of this study, the peak strain was within the normal standing physiological region [47].

In conclusion, regarding the validity of the predicted results of the geometric and mechanical characteristics of the finite-element foot–shoe model, the simulation results showed good agreement with the measured results and the results of related studies. The main reason for discrepancies between the measured and simulated results was the contact relationship between the foot and sole. However, if the model was foot plantar–insole full-contact running shoes, which is different from the actual test shoe model, it would be valid and reasonable, and be used to simulate and analyze the effects of midsole hardness on the mechanical characteristics of the PF.

### 4.2. Biomechanical Mechanisms of the Effects of Midsole Hardness on the PF

A change in the running-shoe midsole hardness changes the sole buckling stiffness. Specifically, buckling stiffness increases with an increase in midsole hardness. Under the same force, an increase in buckling stiffness limits the movement of the MTP joint. Some studies stated that increasing insole hardness could better maintain and support the foot arch [16]. The PF is anatomically located between the calcaneus and the phalangeal joint and the proximal phalangeal bone and under the entire longitudinal arch. The push-off during running causes the joints to repeatedly move, and decreases the arch of the foot. If this occurs for a prolonged duration, chronic strain may be exerted on the PF, which could induce plantar fasciitis. The FE simulation results of Chen et al. [29], and Lin et al. [48] indicated that, at push-off, PF tension increases with an increase in the MTP joint angle. Moreover, experimental cadaver studies by Carlson et al. [10], and Gefen et al. [49] using finite-element simulation and X-ray methods confirmed this conclusion. The results of our study are consistent with these findings: ∠α increases as midsole hardness increases, resulting in a consequent increase in PF stress. We also found that arch height increased as midsole hardness increased. A greater midsole hardness level could support the arch, and arch collapse causes the fascia to be pulled and to bear a higher level of tension [44].

The midsole of running shoes plays a major role in shock absorption during running. Within a certain load range, a harder midsole is associated with worse cushioning performance and greater impact force on the feet [50]. Cheng et al. [51] used an established 2D finite-element model to analyze PF stress under different loading conditions at push-off during running. They found that PF stress was relatively large when the foot was heavily loaded. According to the relevant studies, higher or uneven force on the foot, and high loading rates are important risk factors for fasciitis [6,7,8]. These aspects are consistent with the results of the present study. With the increase in midsole hardness, outsole peak pressure increased, which means that the foot load also increased.

The results of this study have important reference significance for the design of the midsole of running shoes. Higher MH hardness could improve the stability of running and reduce the stress of the plantar fascia. Amateur runners suffering from plantar fasciitis could choose running shoes with higher MH, and manufacturers should consider the MH material as the midsole of running shoes to reduce the risk for plantar fasciitis. Moreover, we will further investigate specific material MH parameters.

### 4.3. Limitation

The present study had several limitations. Several assumptions were made in establishing the foot–shoe model and push-off simulation during running. First, the contact relationship between the foot and the insole was defined using the embedded region constraint. However, according to the validation results of the foot–shoe model, follow-up research results could be considered the simulation results of wearing full-contact foot–insole running shoes [45]. We will try to further refine the contact mode between feet and shoes, such as using full contact for some parts and semicontact for others to produce a model that is closer to reality. Second, owing to the limitations of the experimental conditions, it was impossible to obtain the actual muscle load. The input of some muscle loads was ignored to obtain simplified boundary conditions. The strength of the triceps surae muscle was estimated only by extracting the strength of the Achilles tendon in the sagittal plane of the foot from inverse dynamics data. However, according to EMG data [52,53], when standing with both feet balanced, except for the triceps crus, other foot and lower-limb muscles showed no evident activity, which could be considered a lack of active contraction. Moreover, Simonsen et al. [54], and Croin et al. [55] demonstrated that the triceps crus is the main part that exerts the plantar flexion moment of the foot at push-off during running. Future studies could render the model more accurate by estimating foot muscle strength as a model loading condition with other methods. On the basis of previous research [32], when we selected one participant, we could only reach a conclusion regarding the characteristics of that subject. Due to the errors of different characteristics, more studies with subject-specific models could be conducted.

## 5. Conclusions

The FE foot–shoe model established in the present study was highly similar to the actual foot–shoe geometry, and could reveal an accurate response to the internal mechanical response characteristics between foot plantar–insole full-contact running shoes and the foot. Furthermore, for foot plantar–insole full-contact running shoes, the increase in midsole hardness could reduce plantar-fascia stress and strain, allowing for the foot to bear higher loads. Amateur runners suffering from plantar fasciitis could choose running shoes with a higher MH, and manufacturers should consider MH material as the midsole of running shoes to reduce the risk for plantar fasciitis.

## Figures and Tables

**Table 1:** Material properties of the finite-element model.

Component	Young’s Modulus E (MPa)	Poisson’s Ratio V	Density (kg·m^−3^)	Cross-Sectional Area (mm^−2^)
Bony Structure	7300	0.3	1500	—
Soft Tissue	1.15	0.49	937	—
Ligaments	260	—	937	18.4
Cartilage	1	0.4	—	—
Plantar Fascia	350	—	937	290.7
Upper Part	11.76	0.35	9400	—
Midsole (10)	Third-order Yeoh hyperelastic model (C10 = 0.052; C20 = −0.074; C30 = 0.072)			
Midsole (20)	Third-order Yeoh hyperelastic model (C10 = 0.127; C20 = −0.149; C30 = 0.153)			
Midsole (30)	Third-order Yeoh hyperelastic model (C10 = 0.273; C20 = −0.256; C30 = 0.185)			
Midsole (40)	Third-order Yeoh hyperelastic model (C10 = 0.372; C20 = −0.327; C30 = 0.227)			
Midsole (50)	Third-order Yeoh hyperelastic model (C10 = 0.655; C20 = −0.725; C30 = 0.634)			
Outsole	8	0.47	2300	—
Ground Support	17,000	0.1	5000	—

**Table 2:** Load parameters for the simulation.

Vertical Axis Joint Net Force (N)	Ankle Plantar Flexion Moment (Nm)	Achilles Tendon Arm (mm)	Achilles Tendon Force (N)
−1454	156	54.2	2878

**Table 3:** PF stress and strain at different levels of midsole hardness.

PF	Stress (MPa)					Strain (%)				
Bundles (Shore A)	10	20	30	40	50	10	20	30	40	50
Firs bundle	2.231	2.224	2.213	2.208	2.194	0.637	0.635	0.632	0.631	0.627
Second bundle	1.504	1.499	1.492	1.489	1.48	0.430	0.428	0.426	0.425	0.423
Third bundle	1.348	1.344	1.337	1.334	1.326	0.385	0.384	0.382	0.381	0.379
Fourth bundle	1.044	1.041	1.036	1.033	1.028	0.298	0.297	0.296	0.295	0.294
Fifth bundle	0.754	0.751	0.747	0.745	0.741	0.215	0.215	0.213	0.213	0.212
Sum	6.881	6.859	6.825	6.810	6.769	1.966	1.960	1.950	1.954	1.934

## Data Availability

The data presented in this study are available on request from the corresponding author.
